# Massively parallel digital high resolution melt for rapid and absolutely quantitative sequence profiling

**DOI:** 10.1038/srep42326

**Published:** 2017-02-08

**Authors:** Daniel Ortiz Velez, Hannah Mack, Julietta Jupe, Sinead Hawker, Ninad Kulkarni, Behnam Hedayatnia, Yang Zhang, Shelley Lawrence, Stephanie I. Fraley

**Affiliations:** 1 Bioengineering Department, University of California San Diego, 92093, USA; 2 Electrical and Computer Engineering, University of California San Diego, 92093, USA; 3 Department of Pediatrics, Division of Neonatal-Perinatal Medicine, University of California San Diego and Rady Children’s Hospital of San Diego, 92093, USA

## Abstract

In clinical diagnostics and pathogen detection, profiling of complex samples for low-level genotypes represents a significant challenge. Advances in speed, sensitivity, and extent of multiplexing of molecular pathogen detection assays are needed to improve patient care. We report the development of an integrated platform enabling the identification of bacterial pathogen DNA sequences in complex samples in less than four hours. The system incorporates a microfluidic chip and instrumentation to accomplish universal PCR amplification, High Resolution Melting (HRM), and machine learning within 20,000 picoliter scale reactions, simultaneously. Clinically relevant concentrations of bacterial DNA molecules are separated by digitization across 20,000 reactions and amplified with universal primers targeting the bacterial 16S gene. Amplification is followed by HRM sequence fingerprinting in all reactions, simultaneously. The resulting bacteria-specific melt curves are identified by Support Vector Machine learning, and individual pathogen loads are quantified. The platform reduces reaction volumes by 99.995% and achieves a greater than 200-fold increase in dynamic range of detection compared to traditional PCR HRM approaches. Type I and II error rates are reduced by 99% and 100% respectively, compared to intercalating dye-based digital PCR (dPCR) methods. This technology could impact a number of quantitative profiling applications, especially infectious disease diagnostics.

The rapid and accurate profiling of pathogen genotypes in complex samples remains a challenge for existing molecular detection technologies. Currently, the identification of bacterial infections relies primarily on culture-based detection and phenotypic identification processes that require several days to weeks to complete. The practical application of molecular profiling technology is limited by several factors. To replace culture, molecular approaches must capture an equally wide array of pathogens while also providing specific and sensitive identification in a turnaround time fast enough to impact clinical decision making^1^,^2^,^3^. Studies also suggest that quantification of pathogen load may offer added benefits beyond what culture can offer^4^. However, the number of microbial genomes present in a clinical sample may be extremely low and/or the sample may be comprised of several different microbes. Current bacteria-targeted rapid screening technologies suffer from non-specific hybridization (e.g. microarrays, FISH), non-specific protein signals (e.g. protein mass spectrometry), or limited resolution of species (e.g. nucleotide mass spectrometry)^5^,^6^,^7^. Sequencing with conserved primers targeting the 16S or rpoB genes is the most useful molecular approach for detecting a wide range of bacteria with broad sensitivity, but is a time-consuming process that requires non-trivial technical expertise, computational resources, and analysis time. Moreover, recent studies report that several NGS platforms for microbial detection approach the analytical sensitivity of standard qPCR assays^3^. For applications where turn around time is critical, high-level multiplexing of PCR-based identification strategies remain an active area of research.

High resolution melt (HRM) has gained popularity as a rapid, inexpensive, closed-tube DNA sequence characterization technique. Precisely heating and unwinding post-PCR DNA amplicons in the presence of a fluorescent intercalating dye^8^,^9^,^10^ or sloppy molecular probes^11^,^12^ loss-of-fluorescence melt curves are generated, providing unique DNA sequence signatures. Several researchers have proposed the expansion of HRM into a broad-based profiling technology by preceding it with universal PCR^13^. Priming conserved DNA regions flanking genetic variation sites or mutations, genetic locus sequence differences can be identified by changes in the gene amplicon melt curve signature. This universal HRM technique replaces the need for targeted primers or probes and relies only on the intrinsic melting properties of the amplified sequence. Universal HRM methods have been developed for several applications, including identification of oncogenic mutations^14^, gene methylation patterns^15^,^16^, and bacterial identification^17^,^18^,^19^,^20^,^21^,^22^. We previously advanced universal HRM to enable single nucleotide specificity for the discrimination of microRNA in the Lethal-7 family and for species-level identification of bacteria using the 16S gene^23^,^24^. However, if multiple sequence variants are present, as often occurs in clinical samples, individual sequences cannot be identified in the conventional universal HRM format consisting of a single bulk reaction^13^,^25^. Likewise, although generally reproducible melt curves are obtained, in-run template standards are typically required to overcome run-to-run variability and enable curve matching by user intensive curve identification procedures. These shortcomings have restricted the application of universal HRM to primarily pure homogeneous samples, constrained the breadth of profiling to only a few sequence variants, and limited the technique’s specificity, since single nucleotide changes often manifest as very slight temperature or curve shape changes.

We previously developed an approach called universal digital high resolution melt (U-dHRM) by integrating universal amplification strategies and temperature calibrated HRM with limiting dilution digital PCR (dPCR) in a 96-well plate format^23^. We demonstrated that this approach, in principle, could overcome many limitations of current profiling technologies to achieve single nucleotide specificity, broad-based detection, single molecule sensitivity, and absolute quantification simultaneously. Separately, we’ve developed machine learning approaches using nested, linear kernel, One Versus One Support Vector Machines (OVO SVM) to automatically identify sequences by their melt curve signatures despite inherent experimental variability^24^,^26^. Through these approaches, we’ve shown that U-dHRM is capable of automatically identifying multiple distinct genotypes in a mixture with single molecule sensitivity and single nucleotide specificity. Others have also demonstrated the ability of U-dHRM to sensitively detect rare mutants/variants^27^,^28^ and also novel variants^29^. These findings suggest that U-dHRM has the potential to offer desirable features for several profiling applications that require a combination of speed, sensitivity, quantitative power, and broad profiling ability. However, no platform exists for accomplishing U-dHRM in a high-content format required to reach a clinically relevant dynamic range of detection.

The sensitivity and quantification power of U-dHRM profiling relies on full digitization of the sample, i.e. spreading the sequence mixture across many reactions so each target molecule is isolated from others. Since the process of loading DNA into wells is stochastic at limiting dilutions, the dynamic range of single molecule detection follows a Poisson distribution, requiring the total number of reactions to be approximately 10 to 100 times the number of sequence molecules. That is, the average occupancy (λ) across all reactions must be 0.1 to 0.01 copies of DNA per well. The probability of DNA occupancy in any well, i.e. the fraction of wells having 1, 2, 3, etc. copies, is given by the Poisson probability distribution P = (e^−λ^*λ^n^)/n!, where n is the total number of wells. U-dHRM is currently performed in traditional PCR multi-well plates using HRM enabled qPCR machines. In this format, only about 9 molecules in a sample can be profiled at the single molecule level per 96-well plate (Fig. 1A, left). Therefore, a major challenge to the advancement of HRM-based profiling is the need for an exponential increase in the number of reactions to achieve scalability for realistic sample concentrations. To this end, a microfluidic U-dHRM system could offer the necessary scalability. Although several reports have documented the use of microfluidic chambers or droplets for dPCR, these platforms cannot accomplish U-dHRM. Microvalve-based dPCR devices (e.g. Fluidigm’s qdPCR) do not have high resolution heating blocks necessary for high resolution melt curve generation and moreover are not programmed to capture fluorescence during heat ramping or identify sequence-specific curve signatures. Microfluidic droplet-based digital PCR devices (e.g. Bio-Rad’s ddPCR) perform endpoint PCR detection in a continuous flow format without temperature control, one droplet at a time, which prevents in-situ, real-time monitoring of fluorescence in droplets, as is needed by U-dHRM. To address these challenges, we developed a platform that accomplishes massively parallelized microfluidic U-dHRM and integrated this platform with our machine learning curve identification algorithm. Our technology achieves single molecule sensitive detection and absolute quantification of thousands of bacterial DNA molecules in polymicrobial samples in less than four hours. We show proof of principle in mock blood samples that highly sensitive, specific, and quantitative bacterial identification is achieved in samples containing a high background of human DNA.

## Results

### Digital HRM Device Concept

**Figure 1:**
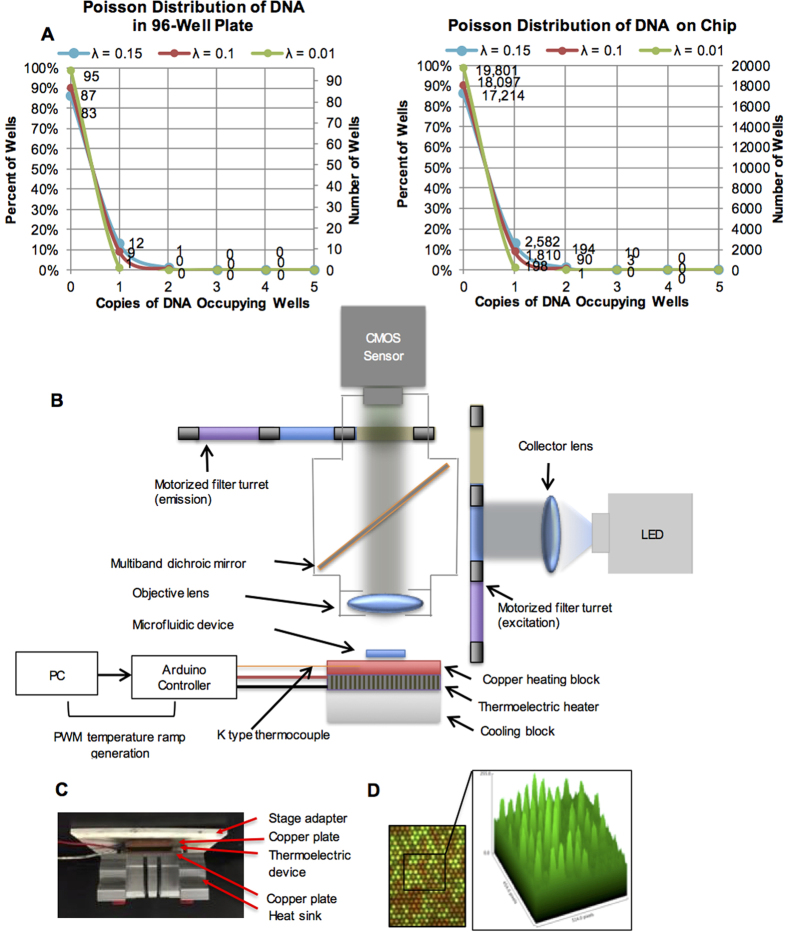
Massively parallel U-dHRM device. **(A)** Poisson distribution of DNA in a 96-well plate versus a 20,000 well digital PCR chip, showing the distribution of molecules per well. **(B)** Schematic of the U-dHRM platform. **(C)** Image of the actual U-dHRM heating setup. **(D)** Fluorescent image of a small portion of chip where background dye (red) and intercalating dye (green) are overlaid. 3D intensity plot of the green channel is shown in inset.

We developed our proof-of-concept U-dHRM platform for the clinical application of neonatal bacteremia diagnosis. Clinically relevant bacterial loads are estimated from culture techniques to be between 1 to ~2,000 colony forming units (cfu) per blood sample (1 ml), where 76% of samples have ≪50 cfu 30,31. This load requires 20,000 reactions to provide a dynamic range of detection up to 1,810 bacterial genomic DNA molecules at the single molecule level (Fig. 1A, right). A digitizing chip fitting this scale of reactions is commercially produced for traditional endpoint dPCR applications (see Methods), and was chosen as a robust and reliable digitizing device. To identify digitized bacterial DNA, universal primers targeting the 16S rRNA gene were used. The 16S harbors conserved sequence regions flanking hypervariable regions that are unique to different genus and species of bacteria^32^. Primers targeting conserved regions generate bacteria-specific amplicons for U-dHRM profiling. Specifically, our long amplicon (~1,000 bp) 16S bulk universal HRM assay^24^ was adapted (see Methods) to enable successful digital amplification and reliable U-dHRM in each of the 725 picoliter volume reactions on-chip, a 99.995% volume reduction compared to the typical HRM reaction format. To enable massively parallel U-dHRM across the 20,000 reactions, we developed a custom high resolution heating device and imaging system. A schematic of our design is shown in Fig. 1B. Precise chip heating was accomplished using a thermoelectric heater/cooler with Arduino controller, power supply, and heat sink. A copper plate was attached between the thermoelectric device and the dPCR chip and between the heat sink and the thermoelectric device to evenly distribute heat. A custom adapter was designed to secure the chip-heating setup onto an automated x,y stage for rapid imaging of the 20,000 reactions as four tiled images at each temperature point during the U-dHRM heat ramp. Figure 1C shows an image of the integrated heating device and stage adapter. The imaging system was equipped with a 4x objective as well as red and green LED-based fluorescence channels. An image analysis program was developed to align reaction well centroids and overcome image drift during heat ramping as well as extract raw fluorescence data from each reaction simultaneously (Fig. 1D). Our previously developed OVO SVM algorithm was adapted to classify and quantify U-dHRM curves after being trained on melt curves generated on-chip. The digital chip, chip heating device, fluorescent imaging system, control electronics, and analysis algorithms for image processing and melt curve identification were integrated to enable massively parallel U-dHRM and absolutely quantitative bacterial profiling.

### System Characterization and Optimization

**Figure 2:**
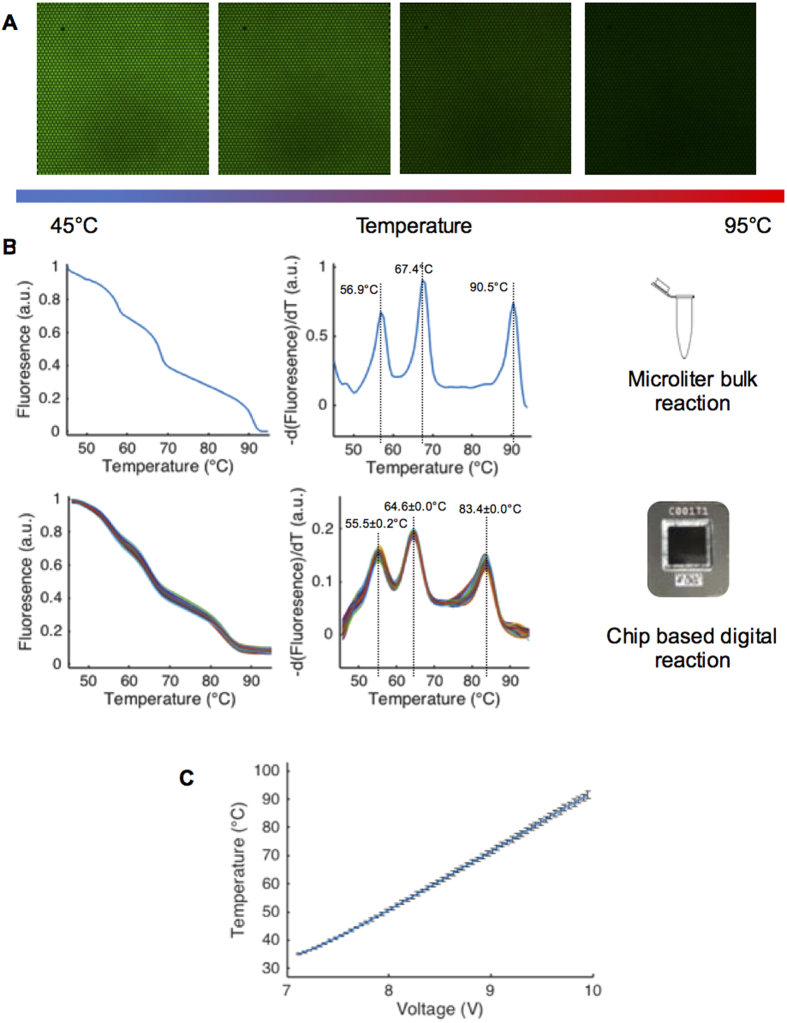
On-chip U-dHRM process characterization and optimization. **(A)** Image of a portion of a chip, which has been saturated with synthetic DNA such that nearly all wells exhibit green fluorescence of intercalating dye. Upon controlled heating, fluorescence is lost as DNA denatures. **(B)** Melting of three synthetic temperature calibrator sequences (pre-made and applied in high concentration to the chip, not PCR amplified) containing different GC content. Optimized ramp rate on-chip compared to bulk qPCR HRM. The mean and standard deviation of the calibration sequence melt curves are shown. **(C)** A plot of the relationship between voltage and temperature for 5 runs, showing it remains linear throughout the HRM temperature range of interest. Standard deviation reaches a maximum of 1.22 °C at 91.6 °C.

The challenge of generating high quality U-dHRM curves in picoliter-scale reactions was first approached by tuning fluorescent intercalating dye concentrations to maximize signal-to-noise ratio. An EvaGreen dye concentration of 2.5X was found to be the highest concentration that did not inhibit amplification on-chip. Next, the simultaneous imaging and heating process of melt curve generation (Fig. 2A) was tuned using three synthetic DNA sequences containing 0% GC, 12% GC, and 76% GC with different predicted melting temperatures (Tms) (Fig. 2B). The greater the GC content, the higher the temperature required to melt the DNA due to higher bond strength. After loading mixtures of these three sequences onto a chip, we performed preliminary calibrations of our device, optimizing imaging exposure time to minimize photobleaching while maintaining the highest possible signal-to-noise ratio. We also used these initial readings to develop our image analysis algorithm (see Methods). Figure 2B shows the normalized fluorescence versus temperature and derivative melt plots for the three calibrator sequences in traditional qPCR HRM and U-dHRM formats. The temperature calibrators are predicted to melt at 57.3 °C, 62.8 °C, and 92.9 °C by melt curve prediction software, uMELT^10^. The average Tms given by qPCR HRM were 56.9 °C, 67.4 °C, and 90.5 °C, respectively, while U-dHRM Tms were 55.5 °C, 64.6 °C, and 83.4 °C. These readings indicated that further temperature ramp optimization was necessary. Improved temperature resolution was achieved by varying the heating ramp rate until a linear and repeatable relationship between voltage and temperature could be maintained throughout our temperature range of interest, 45–95 °C. For highest accuracy, temperature was monitored during the ramp by placing a thermocouple inside a surrogate oil-filled chip and placing this chip next to the calibrator loaded chip. A ramp rate of 0.02 ^o^C/sec was found to give optimal linearity and repeatability of the voltage and temperature relationship, with maximum standard deviation of 1.22 °C occurring at a temperature of ~91.6 °C over 5 runs (Fig. 2C).

**Figure 3:**
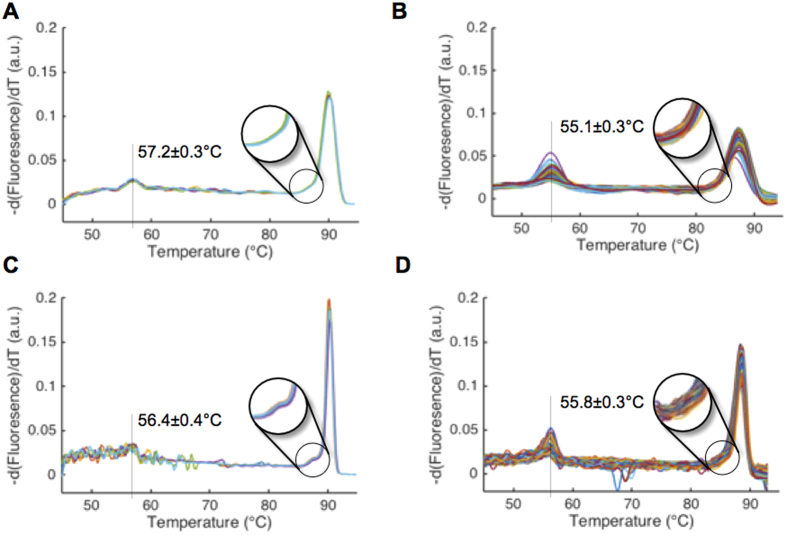
U-dHRM sampling and ramp rate optimization on-chip. **(A,B)** *L. monocytogenes* melt curves generated with a low imaging rate on qPCR HRM and U-dHRM platforms respectively. **(C,D)** *L. monocytogenes* melt curves generated using a high imaging rate on qPCR HRM and U-dHRM platforms respectively. The synthetic temperature calibrator sequence mean melting temperature and standard deviation are shown in all. Black circle highlights a melt curve shape feature unique to *L. monocytogenes* 16S sequence, which is dependent on sampling rate.

**Figure 4:**
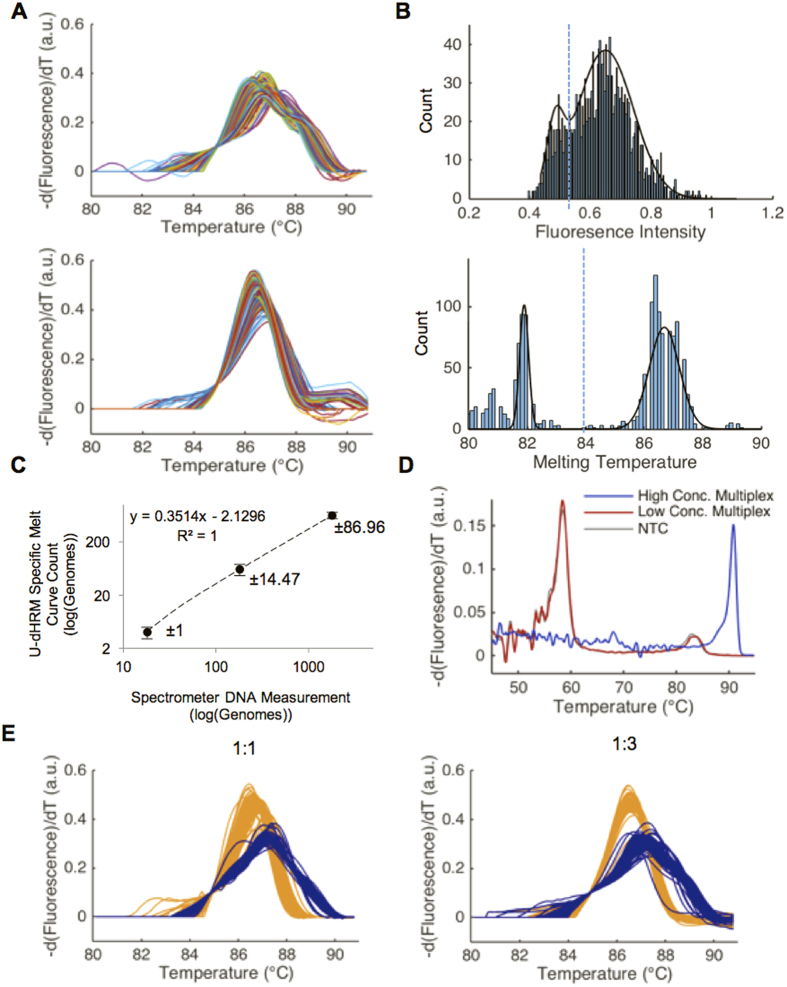
OVO SVM classification of *L. monocytogenes* and *S. pneumoniae*. **(A)** Two-thousand normalized *S. pneumoniae* **(top)** and *L. monocytogenes* **(bottom)** U-dHRM melt curves aligned to 0.1 –dF/dT, respectively. These curves were used to train the OVO SVM to classify each bacteria. **(B)** Histogram of fluorescence intensity values of digital reaction wells with PDF overlay and the intensity value chosen to classify positive from negative marked by dotted line (top). Histogram showing the Tm of each digital reaction with PDF overlay and the Tm value chosen to classify positive from negative marked by dotted line (bottom). Both graphs correspond to a concentration of 458 genomes of *L. monocytogenes* per chip. **(C)** U-dHRM dilution series of *L. monocytogenes* with U-dHRM measured values plotted against spectrometer measured values for DNA content. The sample mean and sample standard deviation are shown. **(D)** In blue: qPCR melt curve generated from a 1:1 mix of 20 ng total DNA input of *S. pneumoniae* and *L. monocytogenes*. In red: qPCR melt curve generated from a 1:1 mix of 0.02 ng total DNA input of *S. pneumoniae* and *L. monocytogenes*. This concentration and reaction mixture is similar to that used for digital chip experiments. In grey: qPCR melt curve generated from a negative template control (NTC) with no bacterial DNA added. **(E)** U-dHRM and OVO SVM classification of *L. monocytogenes* and *S. pneumoniae* in two distinct mixture compositions, demonstrating polymicrobial detection capability. Table 2 shows enumeration of detected curves in panel E.

Next, bacterial DNA from clinical isolates of *Listeria monocytogenes* and *Streptococcus pneumoniae*, two common pathogens causing neonatal bacteremia^33^, were used to further optimize signal-to-noise ratio and melt curve shape resolution (i.e. temperature resolution). First, HRM optimization was carried out on a standard qPCR HRM machine. In this format, melt curve shape, a key discriminating feature of bacterial 16S melt curves^24^, was found to be highly dependent on imaging rate. A low imaging rate of 1 image per 0.3 °C smoothed melt curve shape features (Fig. 3A, circle), but a faster imaging rate of 1 image per 0.1 °C captured small shape differences known to be identifiable by our machine learning algorithm^24^ (Fig. 3C, circle). Using the optimized chip heating ramp rate described above, we next optimized imaging rate on the standard qPCR HRM machine and validated these settings on our U-dHRM system (Fig. 3B and D). The low calibrator sequence (first peak from left in Fig. 3 melt curves) was included in all amplification reactions to align curves and overcome temperature variation across reaction wells. First, the chip imaging rate was adjusted to replicate the default qPCR machine of 1 image taken every 0.3 °C. Imaging the chip every 15 seconds at the optimal heat ramping rate of 0.02 °C/sec on our U-dHRM platform allowed us to achieve this rate. Melt curves generated from these settings constitute the low imaging rate data in Fig. 3B. With these settings, the average peak-to-baseline ratio of the 16S amplicon derivative melt curves (after min-max normalization of raw melt data) was 0.1096 ± 0.0024 on the qPCR HRM machine versus 0.0660 ± 0.0034 for U-dHRM. We then increased the imaging rate on our U-dHRM system to image every 5 seconds, matching the high imaging rate of 1 image per 0.1 °C on the qPCR HRM machine (Fig. 3D). At the high imaging rate, the average peak-to-baseline ratio of the 16S amplicon derivative melt curves was 0.1759 ± 0.0073 on the qPCR machine versus 0.1225 ± 0.0066 for U-dHRM, demonstrating that our device achieves comparable signal-to-noise performance. Small shape differences in melt curves were also identifiable on-chip but to a lesser degree than in the standard qPCR HRM machine (Fig. 3A–D, circles). However, higher background noise on-chip caused this detail to occasionally be lost during curve processing and normalization (Fig. 4A, bottom). Tm reproducibility was almost identical between the two optimized platforms, as demonstrated by the Tm standard deviation of the temperature calibrator sequence (~0.3 °C, Fig. 3). Because this deviation still existed under optimized conditions, temperature calibrator sequences were included in all reactions for aligning melt curves prior to further analysis.

We then integrated our automated OVO SVM melt curve identification approach with our U-dHRM platform to enable automated identification of bacteria based on their melt curve signatures. A training database of bacterial melt curves was generated on-chip to enable automatic curve identification. Bacterial DNA from *L. monocytogenes* and *S. pneumoniae* were loaded onto separate chips in excess, λ of 223 and 141, respectively, as calculated from spectrometer readings. This ensured each of the 20,000 reactions would be positive for amplification and would generate a training melt curve for the bacterial isolate. Each sample underwent U-dHRM using the optimized ramp and imaging rates described above. Figure 4A shows the U-dHRM training curves generated on-chip for *S. pneumoniae* and *L. monocytogenes* after processing with our image analysis, normalization, and alignment algorithms (see Methods). The processed curves were entered into our OVO SVM algorithm as training data (see Methods). Leave One Out Cross Validation (LOOCV) reached a maximum classification accuracy of 99.9% within the training dataset with 1,500 training curves.

### Absolute Quantification of Bacterial DNA

Digital quantitative power relies on the ability to specifically identify true positive amplification from non-specific background amplification. To assess the absolute quantitative power of our platform, we compared U-dHRM melt curve quantification to intercalating dye-based endpoint dPCR quantification. A chip was loaded with a monomicrobial DNA sample of *L. monocytogenes* according to the concentrations described in the lower panel of Table 1 and U-dHRM was conducted. Then, true positive amplification was quantified two ways. For the first quantification method, we followed the typical endpoint PCR enumeration approach (top graph in Fig. 4B), which is based on measuring the fluorescence of all wells at room temperature, fitting the distribution of well fluorescence values to a probability density function (PDF), and applying a fluorescence threshold that best separates the high intensity population (positive) from the low intensity population (negative). For the second method, we used our U-dHRM melt curve readout to identify the number of digital reactions having specific bacterial melt curves. The Tm for a bacterial amplicon, 1,000 bp long, was expected to be centered at 86.5 °C, based on data collected from the overloaded training chips (Fig. 4A). To automate identification of reactions that specifically generated bacterial melt curves, we fit a PDF to the distribution of individual reaction Tm values and applied a fluorescence threshold that best separated the high Tm population (positive, specific amplification) from the low Tm population (non-specific or negative for amplification), shown in the bottom graph of Fig. 4B. This novel analysis is uniquely enabled by our platform. The melt curves identified as positive or negative by this method are shown in Supplementary Fig. 1B and C, respectively. A no template control (NTC) sample was also run on a separate chip to characterize the Tm of non-specific amplification products. The Tm of the NTC chip reactions were significantly lower than the Tm of the 1,000 bp amplicon (Supplementary Fig. 1). Comparable NTC reactions carried out in a qPCR format generated a non-sense amplicon that is 200 bp or less (data not shown). This amplicon size difference is likely the reason for the significant difference in melt curve Tm between the NTC and true positive reactions. The results of the typical dPCR enumeration method and our novel melt curve enumeration method were then compared by direct visual

observation (manual analysis) of the reactions. Visual melt curve observation is used frequently after qPCR to determine whether an amplification reaction was specific or non-specific. This analysis showed that the dPCR enumeration approach gave a Type I (false positive identification of reactions having non-specific melt curves) error rate of 22.6% and Type II (false negative identification of reactions having bacteria-specific melt curves) error rate of 1.19% (average across 3 chips), resulting in a lower limit of detection of ~238 genomes per chip. Our automated melt curve enumeration method based on Tm gave Type I and II error rates of 0.07% and 0.00%, respectively (average across 3 chips) compared to manual analysis, which enables a single copy detection limit. This suggests that our platform could enable general intercalating dye-based dPCR quantification to perform more reliably, even for difficult-to-optimize or partially inhibited reactions that can occur with clinical samples. We then analyzed a ten-fold dilution series of monomicrobial DNA samples of *L. monocytogenes* on-chip using the melt curve enumeration method of Tm thresholding. This showed a linear relationship across the monomicrobial DNA dilution series having an r^2^ value of 1 and high measurement precision demonstrated by the low sample standard deviations at each dilution (Fig. 4C).

Next, we compared the number of curves quantified by our melt curve Tm enumeration method with the sample DNA concentrations calculated from spectrometer readings and qPCR standard curve methods (Supplementary Fig. 2). Table 1 shows that our U-dHRM platform and melt curve enumeration method detects *total* DNA concentrations at similar levels as the other two technologies. However, our approach suggests that U-dHRM is able to distinguish *target* DNA from *background* amplified DNA based on melt curve Tm.

### Identification and Quantification in Polymicrobial Samples

To begin to test the specificity and breadth of profiling of our U-dHRM platform, mock polymicrobial samples were generated to represent challenging detection scenarios where one organism vastly outnumbers another. Defined mixtures of *S. pneumoniae* and *L. monocytogenes* DNA were prepared at two different ratios, 1:1 and 3:1, respectively (Table 2). These mixtures were applied separately to two chips at concentrations nearing the low and high end of a typical clinical pathogen load for neonatal bacteremia (50–2,000 copies). Importantly, this dynamic range cannot be assessed by any current HRM format (Fig. 1A). The heterogeneous samples were subjected to U-dHRM followed by automated Tm thresholding for true-positives and subsequent OVOSVM analysis. Figure 4E shows the OVO SVM identified melt curves for the 1:1 and 1:3 ratios, respectively. Yellow melt curves represent those identified as *L. monocytogenes* and blue as *S. pneumoniae*. Table 2 displays the bacterial composition of the sample reported by the OVO SVM output, i.e. total number of curves classified into each bacterial identity category. The same 1:1 mixture was analyzed by qPCR HRM for comparison, (Fig. 4D). Bulk qPCR HRM fails to indicate the presence of two distinct bacterial species (blue curve) or, in cases of very low DNA input, the presence of any bacteria at all (red curve) due to overwhelming background amplification that results in a melt curve matching the NTC melt curve. This is a common problem for PCR reactions involving universal bacterial primers, since fragments of contaminating bacterial DNA are often present in reagents and liquid handling disposables^34^,^35^. Extensive pre-treatment of all reagents and supplies with DNase can help to improve this. However, contamination of the actual sample cannot be dealt with in the same way, and must be overcome by the detection methodology.

### Detection and Quantification of Microbial DNA in Mock Clinical Samples

**Figure 5:**
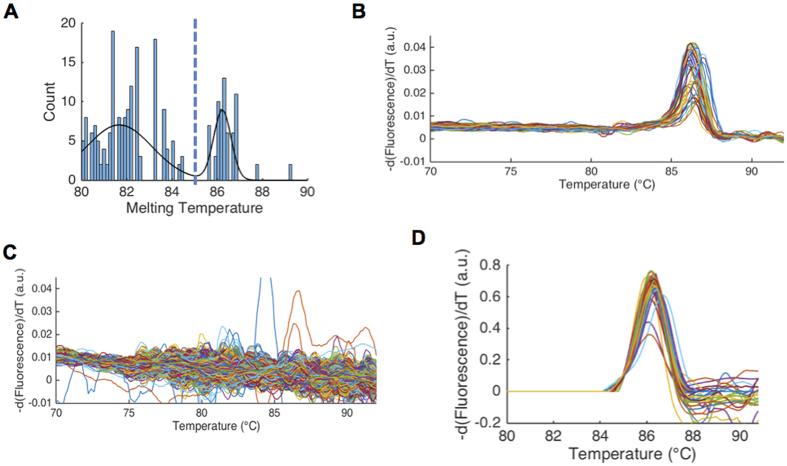
Identification of *L. monocytogenes* in mock blood sample. **(A)** Histogram showing the Tm of each digital reaction with PDF overlay and the calculated Tm threshold (dotted line) used to classify true positive from off-target amplification. **(B)** Bacterial DNA melt curves from reactions identified as positive using the Tm and peak height thresholds adjusted for human DNA background. **(C)** Melt curves from reactions identified as negative using thresholds specific for human DNA background. This plot highlights the high background noise associated with the addition of human DNA to our sample **(D)** *L. monocytogenes* melt curves from panel B normalized and aligned to 0.1 − dF/dT.

A mock experiment was conducted to test whether the large amount of human DNA associated with a clinical blood sample would inhibit U-dHRM pathogen identification. Human DNA, extracted directly from a clinical blood sample of a healthy patient, was mixed with DNA from *L. monocytogene*s in the range of a typical pathogen load (<2,000 bacterial genomes/ml blood). This mixture was loaded onto the chip and U-dHRM was performed using our integrated platform. A Tm threshold value was calculated (Fig. 5A) for separating reactions positive for bacterial amplicons (Fig. 5B) from negative reactions (Fig. 5C). This Tm threshold was higher than the one calculated previously for bacteria-only samples due to a distinct background amplification profile, presumably originating from the human DNA. Human DNA background was associated with more noise in non-specific melt curves, as shown in Fig. 5C, compared to samples that did not include human DNA (Supplementary Fig. 1C). This higher level of noise resulted in slight adjustments to the threshold values used to delineate background from true melt curves (Fig. 5B and C, also see Methods). Nonetheless, 121 *L. monocytogenes* genomes per 20,000 reactions were identified. Figure 5D shows the bacterial melt curves identified in the mock clinical sample by our U-dHRM platform with automated analyses.

## Discussion

Our integrative U-dHRM platform advances HRM profiling by enabling the absolute quantification and identification of multiple genotypes in heterogeneous samples and at clinically relevant concentrations. By achieving HRM curve generation in 0.005% of the traditional HRM volume, and by massive parallelization of HRM across 20,000 reactions simultaneously, we achieve over a 200-fold increase in the dynamic range of detection compared to current HRM formats. Reduction in the size of reactions allows smaller volumes of reagents to be used while maintaining optimal reagent concentrations. Partitioning heterogeneous mixtures across 20,000 picoliter-scale reactions is also expected to overcome environmental microbial DNA contamination that may occur in real-world samples by spatially diluting, i.e. contaminating DNA and target DNA are partitioned from each other for discrimination and quantification^23^. An increased number of reactions also permits rapid generation of a large training curve database for each organism. Incorporating reference temperature calibrator sequences into each reaction helps normalizes against reaction condition variations for improved reliability. Automated melt curve identification is accomplished by removing non-specific melt curves by Tm thresholding and subsequently matching the remaining melt curves to a training database using our OVO SVM machine learning algorithm^24^,^26^. Together, these approaches comprise our microfluidic U-dHRM system and enable the quantitative characterization of complex samples containing multiple bacterial organisms.

Intercalating dye-based dPCR is typically used to detect a single, specific amplification product from one bacteria. Probe-based dPCR can be used to specifically identify up to four bacteria by multiplexing fluorescent probes, or a universal probe can be designed to detect the presence of bacteria non-specifically. By incorporating HRM and universal amplification into dPCR, our platform enables probe-free differentiation of multiple bacteria in a single sample. In our previous work, we showed that 37 clinically relevant organisms could be distinguished by general intercalating dye-based melt curves^24^. We anticipate that our U-dHRM platform will achieve at least this level of multiplexing and potentially more, since we were able to accomplish a signal to noise ratio and temperature resolution on-chip that matched standard qPCR HRM machines.

While a direct comparison of our U-dHRM detection method to a universal probe-based dPCR detection method was not feasible, due to different polymerase and reaction chemistry requirements, a comparison to typical intercalating dye-based dPCR techniques suggested that our platform and automated analysis approach may offer specificity and sensitivity improvements. Standard intercalating dye-based dPCR relies on thresholding total fluorescence intensity of digital reactions to determine whether they are positive or negative for amplification. Inhibitors that reduce amplification efficiency or non-specific background amplification could result in fluorescence intensities that are misclassified, giving rise to false positives and false negatives. However, melt curve analysis may offer a more reliable way to resolve these two conditions. For our reaction chemistry, we found that the typical dPCR approach of applying an intensity threshold to remove false positives left a significant number of reactions misclassified. Bacteria-specific melt curves were observed in several reactions classified as negative by this technique, and non-specific melt curves were observed in several reactions classified as positive. Our platform enabled Tm thresholding, which improved accuracy by 99% and 94%, respectively, in the Type I and II error rates based on manual observation of melt curves. Our approach could help to ensure that true single molecule sensitivity is attained for optimal lower limit of detection. One reason dPCR total fluorescence thresholding performed poorly in our study could be that we thermocycled significantly longer than most dPCR protocols recommend. A typical dPCR cycle number is kept to ~35, but we find that 70 cycles ensures full endpoint amplification from single molecules^23^. While this extended cycling improves accuracy of single-molecule target detection, it also allows off-target amplification to fluoresce more prominently in negative reactions.

Indeed, U-dHRM experiments showed evidence of two kinds of non-template amplification: non-template bacterial DNA amplification (contamination) and off-target amplification. Bacterial contamination produced distinct melt curves within the Tm range of 84–90 °C (Suppl Fig. 1C). Given their high Tm, these melt curves are only likely to arise from amplification of the bacterial 16S gene long amplicon (~1 kbp). Sources of bacterial PCR contamination, which broad-based 16S amplification is highly sensitive to, include molecular biology grade water, PCR reagents, the environment, and DNA extraction kits^36^. Many studies have identified DNA polymerase preparations as the primary source of PCR contamination. The contamination of commercially available polymerase preparations is estimated at 10–1000 genomes/U enzyme^37^. Thus for our system, we would expect between 2.9 and 290 contaminating bacterial genomes per reaction, which is consistent with our observations (Suppl. Fig. 1C).

Off-target amplicons were observed to melt at lower temperatures (Suppl. Figs 1C and 4D, Tm of ~81 °C). In U-dHRM, these products only arose in wells that were negative for bacterial DNA (Suppl. Fig. 1B and C). In qPCR, this off-target product was present in low-template and water control reactions and out-competed bacterial DNA in these conditions (Fig. 4D and Suppl. Fig. 3). Based on Sanger sequencing analysis, this amplification product was non-specific (data not shown) and ~150 bp long by gel electrophoresis analysis (Suppl. Fig. 3). Low reaction efficiency associated with long amplicon PCR and increased cycling time likely contributed to this non-specific amplification. An amplicon size <200 bp is ideal for qPCR. However, our goal is to discriminate numerous bacteria by their 16S sequences, where hypervariability occurs over ~1 kbp. Thus, for specific bacterial identification, we require a 1000 bp amplicon, which can reduce qPCR efficiency significantly^38^,^39^. In highly efficient qPCR reactions, unintended amplification products usually amplify at a lower efficiency than that of the target, and so are out-competed. However, long amplicon targets suffer from low amplification efficiency^38^, allowing off-target amplification to more readily overtake target amplification when the amount of template is relatively low. This reduces the sensitivity of qPCR assays for low-level targets. Our standard curves show that we experience low amplification efficiency comparable to that reported by others in the literature (e.g. 60%, Suppl. Fig. 2A)^39^. This explains the poor sensitivity of qPCR to low target concentrations (Suppl. Fig. 2A).

Importantly, it also highlights a strength of U-dHRM. Because digital reaction partitioning (1) reduces the effect of inhibitors, (2) reduces the effective concentration of contaminating DNA molecules that give rise to off-target amplification, and (3) allows for extended cycling to overcome low efficiency of amplification, since quantification is an endpoint measurement, it is not surprising that we achieve greater sensitivity in the dHRM format (Fig. 4C) than in a qPCR format (Suppl. Fig. 2A). Critically, our integration of HRM with dPCR allows for detection of target, contaminant, and off-target amplification products, and our OVO SVM approach for melt curve signature identification and quantification enables broad-based, automated identification of bacterial organisms.

However, some foreseeable limitations exist. Improvements to the temperature ramp reliability will be critical to ensure a larger database of melt curves are reliably resolved by U-dHRM. Here, calibrator sequences were used to align curves for initial Tm thresholding, but subsequently aligned to their derivative fluorescence value of 0.1 for shape comparison. This second alignment had the effect of ignoring Tm differences in bacteria-specific amplicons, and was required due to fluctuations in the temperature ramp from run-to-run. Insulation from environmental temperatures, an improved chip design with lower thermal mass, and incorporation of a PID controller are expected to overcome this issue. These improvements could also to lead to reduced background noise in the melt curve signal. This would improve our ability to resolve small changes in melt curve shapes generated on the U-dHRM platform, which are occasionally removed by our curve processing algorithms due to background noise.

The capabilities of our microfluidic U-dHRM system could impact infectious disease detection applications like neonatal bacteremia, where speed, breadth of detection, and sensitivity are critical factors. Clinical microbiology relies on lengthy culture-based assays to diagnose bacteremia, which has a high mortality rate that increases with every hour a patient goes undiagnosed and imprecisely treated. Polymicrobial bacteremia is associated with an even higher mortality rate than monomicrobial infection, highlighting the need to detect multiple organisms sensitively, and simultaneously. Immediate conservative treatment with broad-spectrum intravenous antibiotic therapy is typically initiated without any diagnostic information, leading to inaccurate and overtreatment as well as misuse of multiple antibiotics giving rise to the emergence of drug resistant pathogens. The ability to identify bacterial organisms in a blood sample within hours could change clinical practice by providing diagnostic information in time to alter treatment decisions. Retrospective studies also suggest that absolute quantification of bacterial genomic load in patients may be useful to assess severity of infection and to predict prognosis^4^. The detection of microbial DNA in clinical samples is typically challenged by the excess of human DNA compared to pathogen DNA, which can contribute to PCR reaction inhibition^4^,^40^,^41^,^42^. DPCR has been shown to decrease the impact of inhibitory substances^43^. Likewise, we find that U-dHRM detection of microbial DNA in mock blood samples is not inhibited by high human DNA background or inhibitors carried over in the DNA extraction from blood. This suggests that our device could have exciting implications in the clinical setting. Future work will focus on optimizing and validating our U-dHRM technology on patient-derived clinical samples.

Finally, computational approaches for anomaly detection are being explored by our group to identify bacterial melt curves that are not represented in our database. Currently, a 16S amplicon that melts above the Tm threshold will be automatically classified by our OVO SVM as the organism to which the melt curve is most closely matched. For undefined samples, where significantly more organisms may arise and unexpected emerging pathogens could be present, it will be crucial to identify whether a melt curve is a poor match to the database curves. Indeed, other groups have discovered new species of bacteria by observing alterations in bulk HRM curves by eye^29^. Automation of this ability would represent a significant advancement for HRM profiling technology and is under development by our group.

## Methods

### High-Content U-dHRM Chip

In order to achieve high-content digital partitioning, the sample is loaded into a commercially available QuantStudio 3D Digital PCR 20 K Chip v2 (Applied Biosystems, Foster City, CA). The chip contains 20,000 picoliter-scale wells manufactured from silicon with a hydrophilic treatment that allows high efficiency sample loading. A PCR-grade oil is deposited onto the loaded chip to prevent sample evaporation during cycling. The chip is sealed with an adhesive lid containing an optical window, which allows for imaging and the generation of melt curves. We chose to use a commercially manufactured chip for performance reliability. We coupled the dPCR chip to our custom designed master mix. The master mix is optimized to consistently amplify full length ~1,000 bp templates of the 16S gene, hypervariable regions V1-V6, and produce high fluorescence signal intensity for melt curve analysis while maintaining optimal surface tension for easy loading. An MJ Research PTC-200 Thermal Cycler (MJ Research Waltham, MA) is used for endpoint amplification. The thermal cycler is tilted at a 30-degree angle to collect the bubbles generated at high temperatures in the PCR-oil. These bubbles are trapped in an air pocket located outside of the chip’s sample region, preventing sample evaporation from the small volume reactions.

### Bacterial DNA Isolation and PCR

Wizard Genomic DNA Purification Kit (Promega Corporation, Madison, WI) was used to isolate DNA from an overnight culture of bacteria, and diluted in PCR water to the desired concentration. Absorbance measurements were made on stock DNA at concentrations within the working range of the spectrophotometer. Then, the DNA was serially diluted, and the expected concentration of the dilution used for dHRM was reported in the Tables and Figures for direct comparison of the different measuring modalities. The optimum PCR master mix for chip amplification, contained in a 14.5 μL reaction, was found to be 1X Phusion HF Buffer containing 1.5 mM MgCl2 (Thermo Fisher Scientific, Waltham, MA), 0.15 uM forward primer 5′-GYGGCGNACGGGTGAGTAA-3′ (Integrated DNA Technologies, Coralville, IA), 0.15 uM reverse primer 5′-AGCTGACGACANCCATGCA-3′ (Integrated DNA Technologies, Coralville, IA), 0.2 mM dNTPs (Invitrogen, Carlsbad, CA), 2.5X EvaGreen (Biotium, Freemont, CA), 2X ROX (Thermo Fisher Scientific, Waltham, MA), 0.02 U/μL of Phusion HotStart Polymerase (Thermo Fisher Scientific, Waltham, MA), 1 μL of sample, and ultra pure PCR water (Quality Biological Inc., Gaithersburg, MD) to bring the total volume to 14.5 μL. The dPCR chip was cycled on a flatbed thermocycler with the following cycle: an initial enzyme activation (98 °C, 30 s), followed by 70 cycles (95 °C, 30 s, 59 °C, 30 s, 72 °C, 60 s). Temperature calibrator sequences with varying GC content used for system optimization are as follows: 0% GC (TTAAATTATAAAATATTTATAATATTAATTATATATATATAAATATAATA-C3), 12% GC (TTAATTATAAAGGTATTTATAATATTGAATTATACATATCTAATATAATC-C3), and 76% GC (GCGCGGCCGGCACCCGAGACTCTGAGCGGCTGCTGGAGGTGCGGAAGCGGAGGGGCGGG-C3)^23^.

### Chip Heating Device

The U-dHRM device consists of a thermoelectric heating/cooling device (TE Technology, Unc. Traverse City, MI) controlled via an Arduino-based interface that uses pulse width modulation (PWM) to generate a temperature ramp (Fig. 1B and C). The thermoelectric device is in direct contact with a copper plate onto which the dPCR chips coated with a thin layer of thermal paste are clamped. This allows for even heat distribution and optimal surface contact. On the reverse side of the thermoelectric chip, an aluminum heat sink is attached to enable fast excessive heat dissipation. A type K thermocouple (OMEGA Engineering, Stamford, CT) is used to measure the temperature for each image taken during the temperature ramping. The thermocouple is fixed inside a surrogate chip, which is attached alongside the sample chip to the copper plate. The temperature readings are acquired by the microscope imaging software (Nikon NIS-Elements) and are embedded in the image file metadata for offline analysis. The complete chip-heating setup is placed in a custom designed 3D printed stage adapter to securely mount the device on the microscope for imaging.

### Fluorescent Imaging

Fluorescent imaging is accomplished using a Nikon Eclipse Ti platform customized for our dHRM system. A Nikon Plan/Fluor 4X objective with a numerical aperture of 0.13 and a working distance of 16.5X minimizes the number of images and time required to scan the entire chip. A Lumencor SPECTRA X LED Light Engine capable of producing 3–4 W of visible light from 380 nm to 680 nm is used as a light source. Images of the loading control dye, ROX, and melt curve intercalating dye, EvaGreen, are captured with 488/561 nm and 405/488 nm excitation/emission filters using an exposure time of 100 milliseconds. Images are captured every five seconds using a Hamamatsu digital camera, C11440 ORCA-Flash4.0. NIS-Elements software is programmed to automatically image the chip as the heating device ramps using the following workflow: define the capture settings for the ROX and EvaGreen channels, set the stage area to the chip’s sample area, generate points within that stage area to image, and run time lapse to image each location for every time point. A Prior Scientific NanoScanZ (Rockland, MA) motorized stage is used to scan and image the entire chip automatically via the software. For every image, the microscope automatically records the temperature registered by our temperature probe within the metadata of the image. This allows for continuous scanning of the chip and recording of the fluorescence intensity in each well while concurrently heating the chip to generate 20,000 melt curves.

### Image analysis and SVM

#### Fluorescence and Tm thresholding for negative reaction removal

Two approaches to thresholding reaction fluorescence for the identification and removal of negative reactions were compared. The typical dPCR approach of thresholding total reaction fluorescence was accomplished by first plotting a histogram in MATLAB of the total fluorescence intensities at room temperature of all chip reactions. The probability density function (PDF) for a mixture of two normal distributions was then applied to identify negative and positive reaction distributions. A threshold was identified at the lowest point of intensity where the two distributions intersected (Fig. 4B, top). This was performed for each sample type (i.e. DNA extracted from pure bacterial culture versus mock blood sample) to identify the appropriate threshold given unique background distributions.

A second approach was developed to identify a Tm threshold that separated off-target amplified reactions from true positives more accurately. First, raw melt curves were converted to derivative melt curves. On fully loaded chips where all reactions were positive (2 training chips) all reactions contained 16S amplicons, which were observed to melt with an average Tm of 89 °C. On digitized chips (3 chips, testing data), off-target amplicons were observed to melt at much lower temperatures, average Tm of 81 °C, while positive 16S amplicons melted reproducibly in the same range as the training chips. For thresholding analysis, the maximum peak height (Tm) above −d(Fluorescence)/dT = 0.01 was found for each derivative melt curve between the range of 75 °C and 93 °C. Then a histogram of the Tms was plotted in MATLAB, and the PDF for a mixture of two normal distributions was applied (Fig. 4B, bottom). Finally, the Tm threshold was chosen at the minima between the two distributions. Reactions melting below this Tm threshold were identified as negatives, while those melting above the threshold were identified as positives. The Tm threshold for samples of DNA extracted from pure bacterial cultures was identified and held constant at 84 °C.

#### Melt curve data generation

In order to generate melt curves from the acquired fluorescent images of the dPCR chip, we implement an automated image processing algorithm in MATLAB. The algorithm generates a binary mask for each temperature point to identify the centroid corresponding to each digital reaction well in the field. Then records the pixel intensity of the 441 neighboring pixels from the images of both the EvaGreen channel and the ROX channel. Each well’s average pixel intensity is plotted against the measured temperature to generate the raw melt curve. Each melt curve is normalized to the ROX channel to account for any differences due to unequal loading. The Tm threshold described above is then applied to remove negative reactions and all incorrectly identified centroids. A Gaussian filter is then used to smooth the curves and the derivative is taken with respect to temperature to obtain −dF/dT. Finally, the curves are aligned via a temperature independent melt curve alignment at 0.1 −dF/dT. This allowed the differences in melt curve shape to be maximized for later identification using a previously developed OVO SVM algorithm^24^. Briefly, an OVO SVM creates a maximal margin separating hyperplane between two data classes (i.e. melt curve signatures) using the Least Squares Method (linear kernel). OVO SVMs were created for all binary combinations of organisms with the training data generated from melt curves of known origin. During classification, a scoring method is applied and the most frequently called classification is chosen as the final melt curve identity.

### Clinical Blood Sample Purification and Analysis

DNA from a clinical blood sample, which was known to be negative for bacterial infection,was extracted and purified using a High Pure PCR Template Preparation Kit (Roche Diagnostics Corporation, Indianapolis, IN). The purified blood DNA was eluted in a 20 μL volume. DNA of *L. monocytogenes* was isolated using the protocol described above in the methods section. Approximately 2,000 genomes of *L. monocytogenes* were added to the purified blood extraction. The maximum amount of the blood and bacterial DNA mixture (8.63 μL) was added to the PCR master mix. The final mass ratio of human DNA to bacterial DNA on chip was 12,172:1. The master mix was then loaded onto the chip and U-dHRM was performed following 70 amplification cycles. The full chip was imaged as four tiles. Changes were made to the Tm thresholding script to account for the increased and oscillatory noise introduced by the blood DNA extract. First, the peak height for the derivative melt curve was raised to −dF/dT = 0.015 to threshold noisier non-specific melt curves from true bacterial amplicons. Second, a lower threshold for melt curve troughs was added at −0.004 −dF/dT, which aided in removing highly oscillatory and anomalous curves.

### Cell Culture

Clinically isolated *S. pneumoniae* and *L. monocytogenes* were grown separately overnight in Luria-Bertani (LB) broth. Sterile conditions were used to ensure uncontaminated growth of each bacteria.

## Additional Information

**How to cite this article**: Velez, D. O. *et al*. Massively parallel digital high resolution melt for rapid and absolutely quantitative sequence profiling. *Sci. Rep.* **7**, 42326; doi: 10.1038/srep42326 (2017).

**Publisher's note:** Springer Nature remains neutral with regard to jurisdictional claims in published maps and institutional affiliations.

## Supplementary Material

Supplementary Figures 1–3

## Figures and Tables

**Table 1:** Comparison of Genomic DNA Quanitfication Techniques.

Bacteria	Method of Quantification	Number of Genomes/μL
*S. pneumoniae*	Absorbance	5780
	qPCR	6554
	U-dHRM total	5460
	*bacterial melt curves*	*1200*
	*non-template melt curves*	*4260*
*L. monocytogenes*	Absorbance	9160
	qPCR	10839
	U-dHRM total	7580
	*bacterial melt curves*	*2260*
	*non-template melt curves*	*5320*

The concentration of genomic DNA isolated from both *S. pneumoniae* and *L. monocytogenes* was measured using an Eppendorf Biospectrometer, by qPCR standard curve method, and using U-dHRM. Total U-dHRM values are the sum of reactions identified as having specific amplification of bacterial DNA plus the reactions having off-target amplification. Reactions having no amplification, i.e. no melt curve, were classified as true negatives and make up the remainder of the 20,000 total reactions per U-dHRM chip (not represented in this table). QPCR standard curves are shown in Suppl. Fig. 2. Absorbance measurements were made on stock DNA, then the DNA was serially diluted. The calculated concentration of the dilution used on chip is reported here for each measurement modality.

**Table 2:** OVO SVM Classification of Mixed Genomic DNA Samples.

Experiment	Species Mixture	Absorbance		U-dHRM	
		Targeted Ratio of Genomes	Estimated Number of Genomes Added to Chip	Measured Number of Genomes On-Chip	Measured Ratio of Genomes
*1*	*S. pneumoniae*		289	60	
	*L. monocytogenes*	1:1	458	113	1: 1.88
*2*	*S. pneumoniae*		1445	238	
	*L. monocytogenes*	3:1	458	119	2:1

DPCR chips were loaded with polymicrobial samples containing different proportions (ratios) of *S. pneumoniae* DNA to *L. monocytogenes* DNA to mimic challenging detection scenarios where one organism dominates a test sample. The targeted mixture ratios were created based on absorbance measurements of individual bacterial DNA concentrations using an Eppendorf Biospectrometer and then analyzed by U-dHRM and OVO SVM classification.
