# Supplementary material for: Massively parallel digital high resolution melt for rapid and absolutely quantitative sequence profiling
Source: Sci Rep. 2017 Feb 8;7:42326. doi: 10.1038/srep42326 (PMC5296755; doi:10.1038/srep42326)
Supplement: Supplementary Figures 1–3 [file srep42326-s1.pdf]

Supplementary Figures

Massively parallel digital high resolution melt for rapid and absolutely quantitative sequence profiling

Daniel Velez Ortiz[1], Hannah Mack[1], Julietta Jupe[1], Sinead Hawker[1], Ninad Kulkarni[2], Behnam Hedayatnia[2], Yang Zhang[1], Shelley Lawrence[3], Stephanie I. Fraley[1,*]

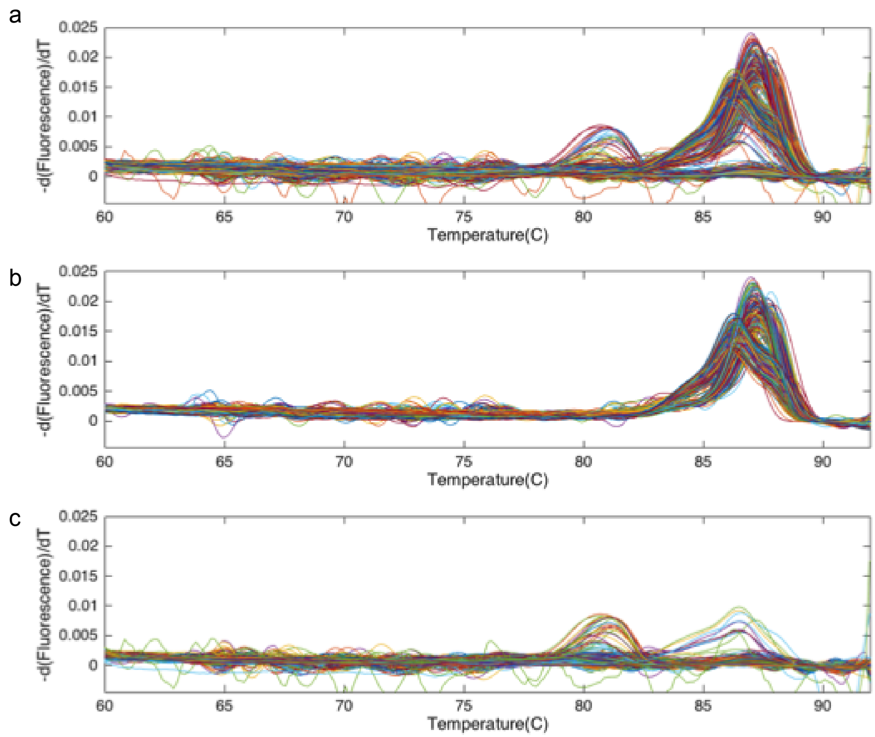

Supplementary Figure 1. DHRM melt curves for polymicrobial mixtures. Curves from experiment 2 in Table 2. (a) All melt curves. (b) Melt curves that were automatically identified as bacterial using our U-dHRM Tm enumeration algorithm. (c) Melt curves that were automatically identified as background/non-specific using our U-dHRM Tm enumeration algorithm.

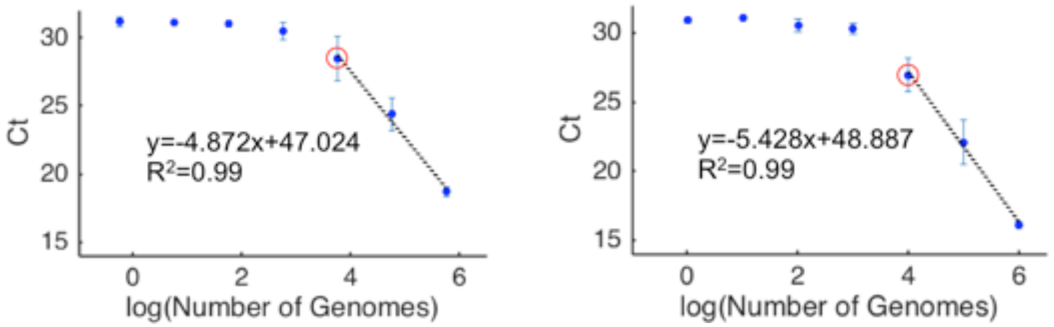

Supplementary Figure 2. QPCR dilution series standard curves. (a) *S. pneumoniae* DNA (b) *L. monocytogenes* DNA. Slope of linear regime indicates primer efficiency. Red circle marks the condition used in the complimentary U-dHRM experiments.

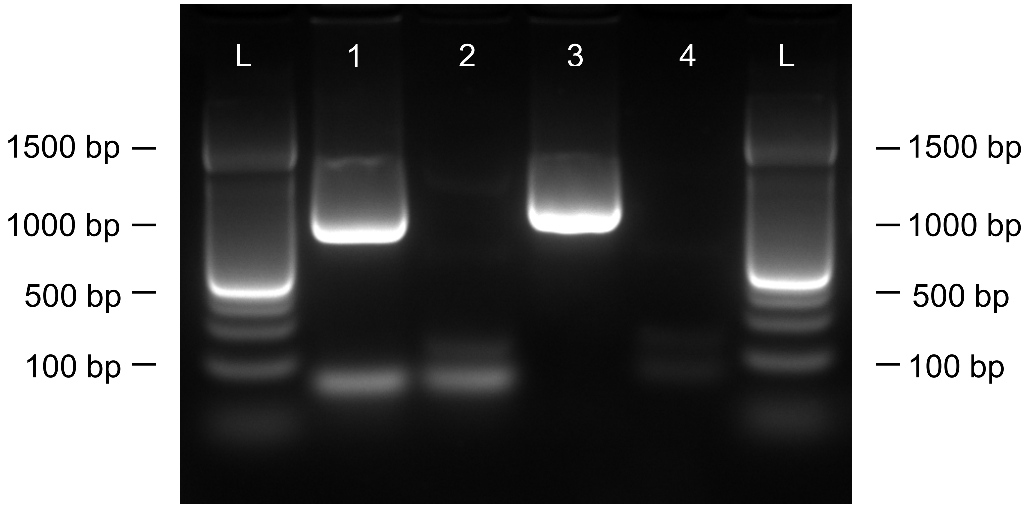

Supplementary Figure 3. Gel electrophoresis of qPCR amplification products corresponding to Fig. 4D. **L:** DNA ladder markers. **1:** High concentration template multiplexed with calibrator sequence. 16S target amplicon appears as a band at 1000 bp. Calibrator sequence appears as a band at 60 bp. **2:** NTC with calibrator sequence, showing a faint off-target amplification band at approximately 150 bp, just above the calibrator sequences. **3:** High concentration template without calibrator sequence included. **4:** NTC without calibrator sequence, showing a faint off-target amplification band at approximately 150 bp.
